# Separation Principles and Strategies for an Oil–Water Separation Membrane with Special Wettability

**DOI:** 10.3390/membranes15080241

**Published:** 2025-08-08

**Authors:** Xiaoying Hu, Tong Xing, Huiyu Wu, Kunyu Wei, Mamadou Souare, Changqing Dong

**Affiliations:** New Energy Generation National Engineering Research Center, School of New Energy, North China Electric Power University, Beijing 102206, China; huxy@ncepu.edu.cn (X.H.); xingt@dteg.com.cn (T.X.); 120242211047@ncepu.edu.cn (H.W.); 120222211005@ncepu.edu.cn (K.W.); 120224300021@ncepu.edu.cn (M.S.)

**Keywords:** superhydrophobicity, superhydrophilicity, oil–water separation, principle, strategy

## Abstract

Although numerous reviews have discussed the research progress in “filtration-type” oil–water membrane separation with special wettability, they predominantly focus on the types of membrane separation and preparation methods, without providing an in-depth analysis of the separation principles and strategies. This paper is different from the previous reviews focusing on the types and preparation methods of membrane separation, mainly as regards membrane surface adsorption, liquid through the pores, and liquid extraction from the pores of the three key nodes in order to analyze the impact of membrane block wettability on the oil–water separation effect of the independent influence. Accordingly, we summed up the membrane separation principle and design strategy to guide modular wettability design during membrane fabrication, thereby enhancing membrane wettability. The modular wettability design approach can provide guidance during the membrane development phase, offering potential solutions to extend membrane lifespan and address issues of surface fouling and pore clogging while enhancing mass transfer efficiency during operation.

## 1. Introduction

Frequent oil spills and the entry of oily wastewater into natural water bodies lead to serious ecological pollution and threaten the health of human beings and other organisms. Among all oily wastewater treatment methods, the separation of oil and water using membrane separation with special wettability is a hot research topic [1].

Wettability is the tendency and ability of a liquid to spread on or adhere to a solid surface. It describes the extent to which liquid molecules (e.g., water, oil) are able to do this. Accordingly, the mechanisms of membrane separation and design strategy can be summed up as guiding modular wettability design during membrane fabrication, thereby enhancing membrane wettability. The modular wettability design approach can provide guidance during the membrane development phase, offering potential solutions to extend membrane lifespan and address issues of surface fouling and pore clogging while enhancing mass transfer efficiency during operation. When a liquid comes into contact with a solid surface, it displaces the existing gas phase (typically air) and spreads across the surface. This fundamental process underlies numerous biochemical reactions. Wettability serves as the determining factor for the oil–water separation performance of membrane materials. The underlying mechanism relies on the regulation of liquid–solid interfacial energy through surface chemical composition and roughness structure, enabling selective wetting behavior to achieve separation.

Inspired by biomimicry, research related to surfaces with special wettability (such as superhydrophilic surfaces with a water contact angle (WCA) close to 0° and superhydrophobic surfaces with a WCA > 150°) has developed rapidly in the last three decades. Jiang et al. [2,3,4] reviewed the research progress on special wettability in detail from the development process of wettability theory, special wettability phenomena in nature, design principles and preparation methods of special wettable surfaces, the classification of special wettability, types of special wettable materials, and applications. Tian and Milionis et al. [5,6], on the other hand, reviewed the assessment of durability of special wettable surfaces and the direction of development from the perspective of wear testing.

Zhang et al. [7] reviewed the research progress on oil–water separation materials from the perspective of separation modes (adsorption and filtration). Adsorption-type materials are suitable for removing floating oil in water. Still, the adsorption capacity is limited, and the desorption process regeneration needs to be processed by washing with organic solvents or centrifugation, which is inefficient and not very environmentally friendly [8,9,10,11,12]. At the same time, membrane separation is suitable for use in continuous filtration separation. Although Qiu et al. [13] reviewed the progress of ‘filtration-type’ oil–water membrane separation in terms of design principles, preparation methods, substrate types, etc., they focused more on the progress of the kinds of membrane separation and preparation methods, and have not yet summarized the separation strategies of oil–water membrane separation. Separation strategy refers to the method of regulating membrane wettability so that the oil and water migrate in distinct ways or directions, thus achieving oil–water separation. This aspect is extremely important but easily neglected in the design stage of the membrane.

In summary, the previous section details various aspects of oil–water membrane separation, and the following summarizes the inadequateness of the above reviews, as shown in Table 1.

**Figure 1:**
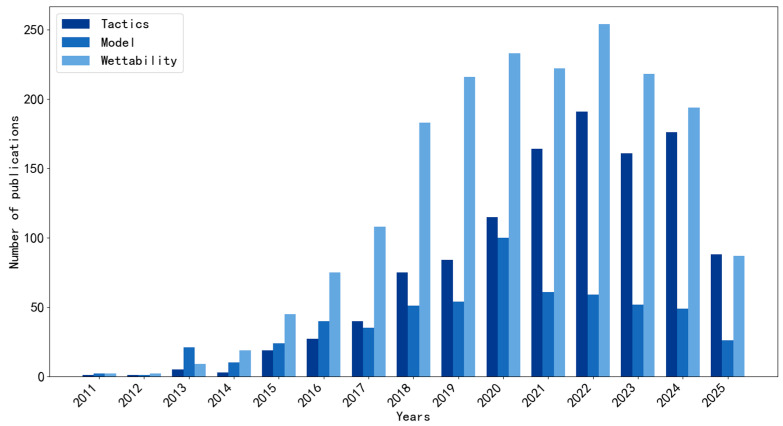
Statistics on the number of relevant papers in the field of oil–water membrane separation, 2000–2025.

In recent years, a statistical search of the SCI database has found a significant increase in the number of related papers (Figure 1), indicating that the field is receiving academic attention. The aim of this paper is to systematically sort out the research results to provide readers with reference and inspiration. This paper focuses on filtration-type oil–water membrane separation and highlights the separation principles and design strategies of these membranes. Firstly, the principles of oil–water membrane separation are summarized from three key processes: adsorption, passage through pores and detachment from pores. Then, the existing separation strategies are classified according to the wettability classification, and the characteristics of different separation strategies are discussed accordingly, so as to provide guidance for the design of membrane separation.

## 2. Separation Principle

The two main factors for the spontaneous selective membrane permeation process of oil and water under gravity are flux and selectivity.

According to the Hagen–Poiseuille equation, the relationship between the volumetric flow rate (Q) and the pore radius (r_p_) for the membrane permeation is given by Q∝$r_{p}^{4}$, assuming all other parameters remain constant. As the pore radius decreases, the viscous resistance to fluid flow through the membrane pores increases, leading to a reduction in flux [14].

(1) $$Q=\frac{\varepsilon \pi ∆pr_{p}^{4}}{8\mu l}$$

In the above equation, ε is the surface porosity; Δp is the pressure difference between the two ends of the fluid; μ is the fluid viscosity; and l is the distance the fluid travels through the surface.

**Figure 2:**
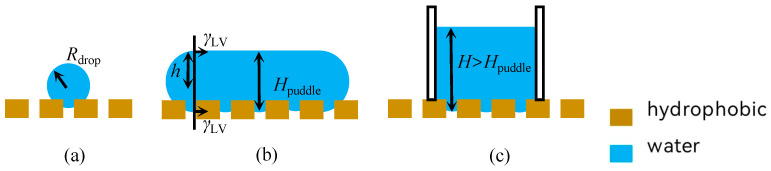
Schematic diagram of the force equilibrium state of different liquid forms on the surface of hydrophobic materials. (**a**) Droplet Modeling; (**b**) Free Liquid Film Model; (**c**) Modeling of Non-Free Liquid Surfaces.

Selectivity is an expression of the equilibrium of forces under the combined effect of the liquid phase’s own gravity and surface tension. The equilibrium/nonequilibrium state of the forces acting on the membrane surface corresponds to the impermeable/permeable state, respectively (Figure 2). The volume of the liquid phase on the membrane surface affects the equilibrium state of the liquid phase. With the increase in the volume of the liquid phase, the equilibrium state of the liquid phase on the membrane surface can be classified into the droplet model (small volume of the liquid phase), the free liquid film model (gradual aggregation of droplets and increase in the volume), and the non-free liquid surface model with a perimeter boundary (further growth of the volume, and elevation of the liquid by the influence of the perimeter) [15].

### 2.1. Droplet Modeling

**Figure 3:**
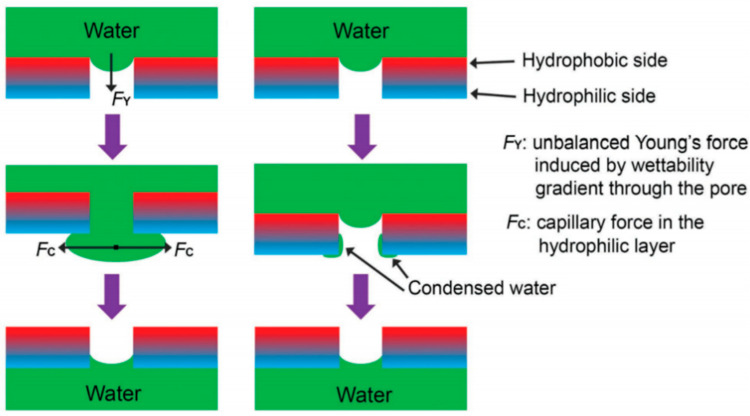
Early hypothesis of directional movement [16]. (**a**) Possible mechanism that water is dragged from the hydrophobic section to the hydrophilic section under the unbalanced Young’s force induced by wettability gradient. (**b**) Possible evaporation-deposition mechanism that water can condensate on the hydrophilic pore surface, and the bridging between the condensed water and the drop will trigger the directional water motion.

**Figure 4:**
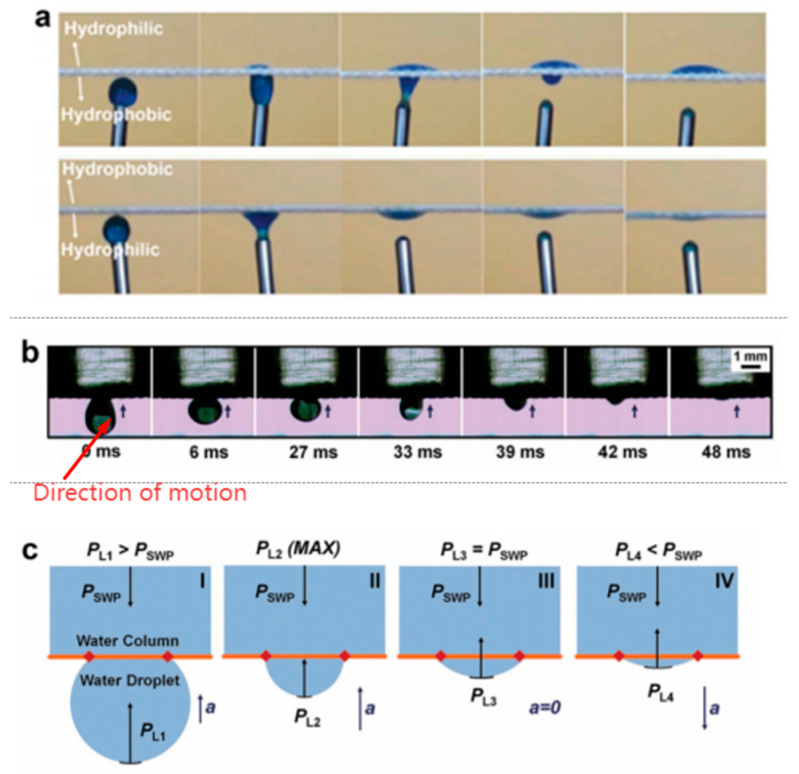
Penetration of droplets from the lower surface of the Janus membrane to the upper surface [18]. (**a**) Directed water transport from the lower hydrophobic side of a hydrophobic/hydrophilic fabric to the upper hydrophilic side, independent of gravity. (**b**) Spontaneous water droplet transport from the lower surface of a superhydrophobic mesh to the upper thin water column. (**c**) Four stages in the droplet ascent process: (**I**) the initial condition; (**II**) the maximum PL; (**III**) the equilibrium of PL and PSWP; (**IV**) insufficient PL for assisting.

The droplet model has been widely used in the unidirectional permeability analysis of Janus membranes (membranes with asymmetric infiltration on both sides) [16,17,18]. Two theories were proposed in early papers: the Laplace force generated by the spherical crown of the droplet, and the drag effect caused by the hydrophobic–hydrophilic gradient on the inner surface of the pore, which drives the droplet to permeate from the hydrophobic to the hydrophilic surface [16] (Figure 3). The first theory only considered the macroscopic droplet curvature, which apparently contradicted the inability of droplets to permeate a hydrophobic membrane with uniform wettability. The second theory only considered the wettability of the microscopic pore surface and neglected the pore properties and the macroscopic effects generated by the droplet surface [19]. Subsequent studies have shown that the gradient in membrane wettability and the Laplace differential pressure at the interface together drive fluid movement (Figure 4) [17,18,19,20].

**Figure 5:**
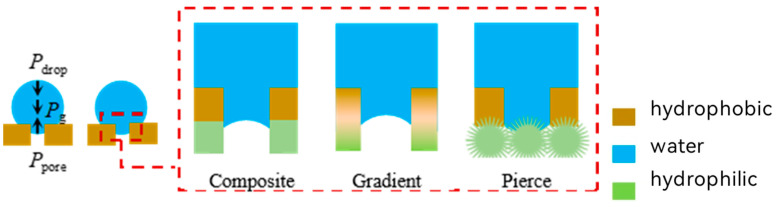
Schematic representation of the forces on a droplet on the surface of a hydrophobic material and typical ways of breaking through the interface within the pore space.

Similarly, the equilibrium state of a droplet on the surface of a hydrophobic film is influenced by the combined effects of wettability and droplet morphology. Droplets form a spherical crown on the membrane surface and a hemispherical interface within the pores. The spherical crown and hemispherical interface generate opposing Laplace internal stresses (Figure 5). The difference in Laplace internal stress (P_pore_ − P_drop_), together with the gravitational force (P_g_), balances to achieve an equilibrium state as described by Equation (2). In this state, the driving force for infiltration is eliminated, preventing the droplets from passing through the hydrophobic membrane. In contrast, through their hydrophobic–hydrophilic gradient, the composite structure of hydrophobic and hydrophilic regions, and the needle-like structures penetrating the liquid membrane, Janus membranes disrupt the hemispherical interface within the pore. This disturbance leads to an inversion or cancelation of P_pore_ in Equation (2), thereby breaking the equilibrium and facilitating infiltration.

(2) $$P_{g}+P_{drop}=P_{pore}$$

In the above equation, P_drop_ is the Laplace force generated by the arc of the spherical crown, P_pore_ is the Laplace force generated by the arc of the hemispherical interface within the pore ($0<\left|P_{pore}\right|\ll \left|P_{max}\right|$), P_max_ is the breakthrough pressure, and P_g_ is the pressure due to gravity.

### 2.2. Free Liquid Film Model

**Figure 6:**
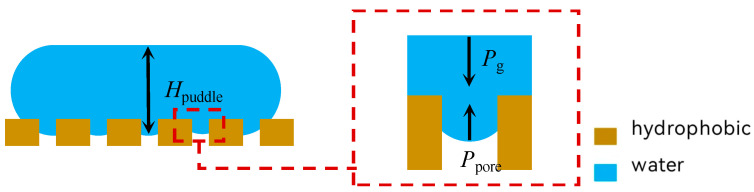
Schematic diagram of the equilibrium state of the free liquid film on the surface of the hydrophobic material.

The free liquid film model is utilized to analyze the reference pressure (minimum pressure) exerted by the liquid on the surface of the film, based on the idealized scenario where the contact angle approaches 180°. As droplets accumulate, they spread across the interface under the influence of gravity and surface tension, forming a liquid film of a specific thickness. At this stage, the interface experiences liquid pressure, as depicted in Figure 6.

(3) $$P_{pudlle}=\rho gH_{puddle}=2\sqrt{\gamma_{LV}\rho g}=\frac{2\gamma_{LV}}{l_{cap}}=P_{ref}$$

Here, P_ref_ is lower than the interfacial pressure generated by a non-free-surface liquid or droplet with a perimeter boundary (i.e., 2R_drop_ << H_puddle_, which is mainly affected by the Laplace force) [21].

The aggregation of droplets on the surface of a hydrophobic film alters the equilibrium state of the droplets, transitioning them to a new equilibrium. As the radius of the spherical crown formed during the aggregation process increases, the Laplace force acting on the left side of Equation (2) decreases. Simultaneously, the Laplace force exerted by the hemispherical interface within the pore, as represented on the right side of Equation (2), also diminishes. This change results in a dynamic equilibrium, whereby the liquid cannot pass through the pore.

### 2.3. Modeling of Non-Free Liquid Surfaces

**Figure 7:**
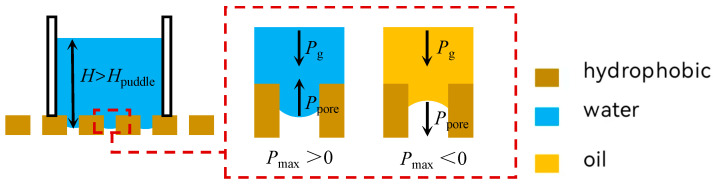
Schematic diagram of the force equilibrium state of a non-free liquid film on the surface of a hydrophobic material.

As the droplets continue to converge, the free liquid film expands to contact the perimeter, causing the spherical crown to disappear. The liquid height increases, and a horizontal surface emerges. However, the meniscus interface within the pore remains intact, as shown in Figure 7. At this stage, the equilibrium expression in Equation (2) transitions to Equation (4). When P_g_ ≪ P_max_, the system remains in dynamic equilibrium, and the liquid phase cannot penetrate. However, when P_g_ > P_max_, equilibrium is disrupted, allowing the liquid phase to penetrate through the pores.

(4) $$P_{g}=P_{pore}$$

(5) $$P_{g}=\rho gh$$

Regardless of the model used, oil–water separation occurs when the permeation process demonstrates selectivity for oil and water. Due to the closer alignment of the non-free-floor model with actual separation conditions and the significant influence of the pore space on the separation process, numerous studies have defaulted to using the non-free-floor model to analyze the semilunar interfacial state of the liquid phase within the pore space. This analysis illustrates the principle of oil–water separation under both gravity-free and gravity-affected conditions [13,21,22,23,24,25]. In the absence of external forces (including gravity), if P_max_ is less than 0, spontaneous permeation of the liquid occurs. Conversely, if P_max_ is greater than 0, the liquid will be impermeable if the pressure is less than P_max_, but permeation will occur if the pressure exceeds P_max_. Under gravitational conditions (without other external forces), the liquid can exert a certain pressure on the membrane surface, with the minimum pressure referred to as the reference pressure, P_ref_. Whether spontaneous permeation can occur depends on the relationship between P_max_ and P_ref_ [21]. Under gravitational conditions, when the robust factor A* (the ratio of P_max_ to P_ref_) is greater than 1, the medium can spontaneously pass through the pore due to gravity. However, when A* is less than 1, additional pressure is required for the medium to permeate the pore [25,26,27].

**Figure 8:**
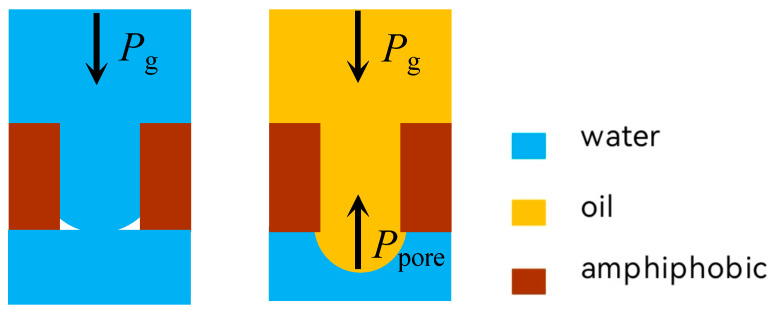
Schematic diagram of the forces on the liquid in the pores of the double hydrophobic material.

Furthermore, the oil–water separation model of the super-double sparse mesh membrane, established and validated by Zhang [28], offers an additional method to enhance the pressure difference required for oil and water breakthrough. By pre-setting an auxiliary screening fluid on the bottom surface of the super-double sparse membrane, the oil–water mixture interface comes into contact with the auxiliary screening fluid under pressure. At this point, the hemispherical interfaces of identical liquids disappear and are free to migrate, while the hemispherical interfaces of dissimilar liquids are retained. Consequently, a significant breakthrough pressure persists, and the migration of liquids is restricted, as shown in Figure 8.

Based on the analysis of the effect of the half-moon interface within the pores on the breakthrough pressure, the membrane can be made oil–water selective by regulating its wettability. In this approach, a superhydrophobic membrane with uniform wettability is used to block water and facilitate de-oiling, while a superhydrophilic membrane is employed to block oil and enable de-oiling. This strategy is simple, cost-effective, and easy to fabricate, making it a prominent area of research. However, the separation of oil–water emulsions is not yet optimal. Droplet modeling can help explain the adsorption of emulsion droplets onto the surface of the membrane, as well as the agglomerations and their effects during the separation process. The principles underlying selectivity in the separation processes—such as liquid-phase adsorption, passage through pores, and detachment from pores—share commonalities but also exhibit distinct differences. These differences can lead to the development of various separation strategies.

### 2.4. Separation Mechanisms

**Figure 9:**
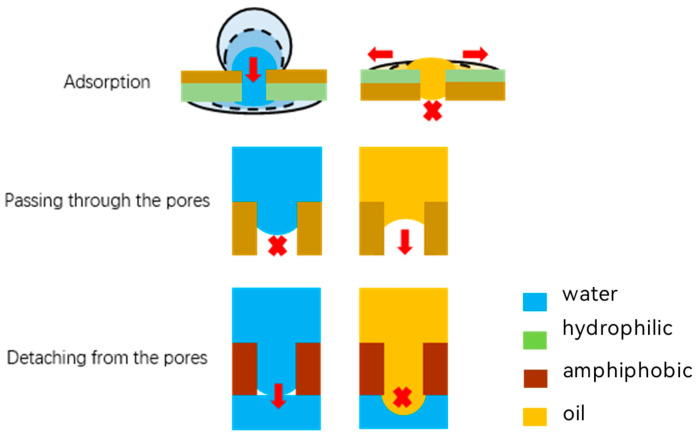
Passage of liquids through porous membranes.

As shown in Figure 9, the design of modular wettability relies on three key nodes in the passage of liquids through membrane separation: liquid-phase adsorption, passage through pores, and detachment from pores. When a liquid mixture comes into contact with a membrane surface with special wettability properties (e.g., hydrophobic, hydrophilic or Janus properties, etc.), the target components are preferentially adsorbed on the membrane surface through physical or chemical interactions (e.g., van der Waals forces, electrostatic attraction or hydrogen bonding, etc.) to form a locally enriched phase (liquid phase adsorption). Driven by pressure, electric field or concentration gradient, the liquid selectively passes through the pore channels (permeation pores) within the membrane that match the wettability, where the modular design of the wettability allows for targeted regulation of the fluid resistance and mass transfer efficiency within the pores. When the liquid flows out of the pore, the liquid phase is detached from the end (detached from the pore) due to the sudden change in interfacial tension, pressure release or fluid shear on the downstream side of the membrane, and the difference in wettability (e.g., hydrophobic–hydrophilic heterogeneous structure) through the modular optimization of pore exit can further regulate the kinetic structure of droplet detachment, achieving efficient separation and collection.

### 2.5. The Infiltration Law of Membrane

(1) Young’s equation and static contact angle

Young’s equation describes the relationship between the static contact angle (θ) and the three-phase interfacial tension based on the mechanical equilibrium of an ideal smooth surface [29]:

(6) $$\gamma_{SV}=\gamma_{SL}+\gamma_{LV}cos\;\theta$$

The size of the contact angle directly reflects the hydrophilicity of the material: θ < 90° is a hydrophilic surface, θ > 90° is a hydrophobic surface, and θ > 150° reaches a super-hydrophobic state [2]. Although the Young equation is an ideal model, it lays the foundation for the establishment of the subsequent rough surface wetting theory [30].

(2) Wenzel’s equation and rough surface correction

The actual surface mostly has a microscopic rough structure, and Wenzel corrects the contact angle by introducing the roughness factor r, which is proposed as follows:

(7) cos θ*= rcos θ

Here, r is the surface roughness factor, the value of which is equal to the ratio of the actual contact area of the material to the ideal contact area (projected area); this parameter always satisfies ≥ 1.

The equation suggests that roughness amplifies the intrinsic wettability of a material: hydrophilic surfaces (θ < 90°) are more hydrophilic and hydrophobic surfaces (θ > 90°) are more hydrophobic [31]. For example, a superhydrophobic stainless steel mesh was surface-coated with PTFE nanoparticles (r > 1), which increased the contact angle from 110° to 156.2° [32], validating the applicability of Wenzel’s model.

(3) Cassie’s Equation and Non-Homogeneous Immersion

The Cassie–Baxter model describes the non-homogeneous immersion state when an air cushion layer is present on a rough surface:

(8) $$cos\;\theta \;*=f_{SL}cos\theta +f_{LV}cos\theta \prime$$

**Figure 10:**
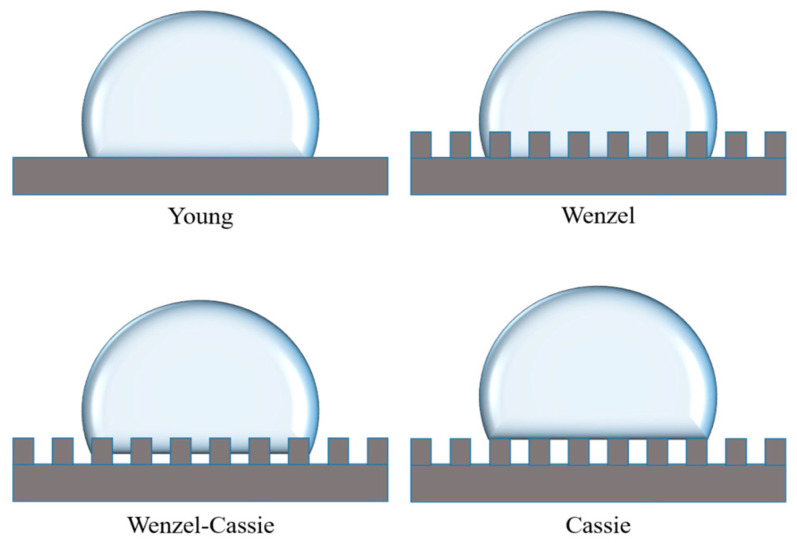
Modeling the wettability of four solid surfaces.

The air cushion layer significantly reduces the solid–liquid contact area, further increasing the apparent contact angle. For example, the micro- and nanocomposite structures on the surface of lotus leaf achieve superhydrophobicity (θ > 150°) by trapping the air layer [32]. The energy barrier between Cassie and Wenzel states determines the wetting stability, and the dynamic external force (e.g., pressure) can induce a transition between the two, which affects the contamination resistance of the membrane separation [33]. The four different models are shown in Figure 10.

(4) Dynamic contact angle and hysteresis effects

The actual infiltration process involves dynamic contact angles, including forward and backward angles. The contact angle hysteresis reflects the obstruction of droplet motion by surface roughness and chemical heterogeneity [33]. Low hysteresis surfaces (Δθ < 10°) favor droplet rolling self-cleaning, while high hysteresis surfaces tend to lead to oil adhesion. For example, Janus membranes were designed to utilize Δθ differences for oil–water directional transport through wettability gradient design, which significantly improved the separation efficiency [34].

## 3. Separation Strategy

Among the various separation strategies, those involving membranes with homogeneous and heterogeneous wettabilities such as superhydrophobic-superoleophilic, superhydrophilic-submerged, superoleophobic, superhydrophilic–superoleophobic materials, and superhydrophilic–superoleophilic (i.e., superbipartite) surfaces are dominant [15,34,35]. Furthermore, the concept of switching wettability between superhydrophobic–superoleophilic and superhydrophilic-submerged superoleophobic surfaces under certain conditions is not fundamentally distinct from these established strategies and principles. Although less research has been conducted on the Janus strategy with inhomogeneous wettability and the superbiphobic strategy with homogeneous wettability, these alternative approaches have introduced novel separation principles, thus expanding the range of separation strategies.

The fabrication strategies for oil–water membrane separation can be generally categorized into two types: one involves building a rough structure on the membrane surface before loading it with low surface energy substances; the second involves creating the rough structure directly with low surface energy substances.

### 3.1. Superhydrophobic–Superoleophilic Strategy

Superhydrophobic membranes with homogeneous wettability are a typical and widely used strategy for oil removal in oil–water separation. Inspired by the superhydrophobic phenomenon observed on the surface of lotus leaves, Feng et al. [1] sprayed a low-surface-energy polytetrafluoroethylene (PTFE) emulsion onto a stainless steel mesh, followed by drying and curing. This process mimicked the “binary synergism” between the micro- and nano-scale structures on the lotus leaf surface and its low surface energy, resulting in the creation of superhydrophobic membrane separation. The superhydrophobic–superoleophilic strategy was then applied to achieve effective oil–water separation. The contact angle of superhydrophobic stainless steel mesh membrane for water and oil (diesel) is 156.2° ± 2.8° and 0°, respectively, which can effectively realize water–oil separation.

Since then, numerous studies have focused on the preparation of oil–water membrane separation based on porous materials such as metals, ceramics, and fabrics using the superhydrophobic–superoleophilic strategy. Rasouli [36] provided a comprehensive review of the modification methods for preparing superhydrophobic–superoleophilic membrane separation from various substrates. However, due to the long-range interaction between the hydrophobic surface and highly viscous hydrophobic contaminants (including oil), membrane separation using the superhydrophobic–superoleophilic strategy is prone to contamination, blockage, reduced service life, and high maintenance costs [37]. Furthermore, during the separation process, oil typically remains in the upper layer of the mixed solution (since its density is usually lower than that of water). The downward movement of oil through the water layer to contact the separation membrane requires additional energy, which is not ideal for oil filtration [38].

### 3.2. Superhydrophilic–Superoleophobic Strategy

Super hydrophilic–super oleophobic strategy is a typical water removal strategy the oleophobic property can make the membrane difficult to block and to contaminate, and the hydrophilic property can make the membrane easy to clean. However, according to the classical theory, because the surface free energy of oil is lower than that of water, the surface energy of hydrophilic material is higher than water, which is inevitably higher than oil, and it will be oleophilic; the surface energy of oleophobic material is lower than oil, which is inevitably lower than water, and it will be hydrophobic. There exists no material that is hydrophilic and oleophobic at the same time. Therefore, the super hydrophilic–super oleophobic strategy is indirectly realized under certain conditions.

#### 3.2.1. Superhydrophilic–Underwater Superoleophobic Strategy

The superhydrophilic–underwater superoleophobic strategy was inspired by the phenomenon of fish scales and mussel submerged oleophobicity, and was realized in a three-phase system of oil, water and solid [39,40]. The membrane water contact angle (WCA) < 5° and underwater oil contact angle (OCA) ≥ 150° using this strategy can block oil and filter water.

The available substrate materials cover metal mesh, fabrics, polymer composite membranes, electrostatically spun membranes, etc. [41,42], and Zarghami [43] summarized in detail the preparation of superhydrophilic–underwater superoleophobic separation molds. Oil–water membrane separation based on this strategy can avoid the adsorption of hydrophobic substances (soluble organic macromolecules and insoluble inorganic substances) on the membrane surface in the aqueous environment due to the underwater oleophobic property [44,45], and can be recycled by simple rinsing. However, it is superhydrophilic–superoleophilic in nature, so the separation of the oil–water emulsion is limited. When individual pores are in an anhydrous environment, oil droplets may then penetrate through the pores, affecting the final separation [44].

#### 3.2.2. Functional Group-Responsive Superhydrophilic–Superoleophobic Strategy

One approach to achieving the superhydrophilic–superoleophobic strategy involves manipulating the arrangement of functional groups on the membrane surface under the influence of water or oil molecules. This alters the surface energy of the membrane, enabling it to exhibit a low water contact angle and a high oil contact angle in air (in air WCA ~0°, OCA ≥ 150°). This strategy results in a membrane that is favorable for water permeation while strongly restricting oil permeation.

Such functional groups typically contain both hydrophilic and hydrophobic elements, such as poly (diallyldimethylammonium chloride)-perfluorooctanoic acid ammonium salt/silicon dioxide (PDDA-PFO/SiO_2_) [46]. These groups preferentially interact with homopolar liquids (e.g., water), while interactions with nonpolar liquids, such as oil, are weak [47]. Fluorine-containing groups are exposed on the outermost layer of the membrane, imparting oleophobicity. The interaction between polar water molecules and the polyelectrolyte on the membrane surface induces a rearrangement of the molecules, positioning the hydrophilic segments at the solid–liquid interface, thereby enhancing hydrophilicity. In contrast, when exposed to oil molecules, the interface is dominated by low surface energy components, and the surface recovers its oleophobicity. However, the time required for this transition is relatively long [26].

### 3.3. Superhydrophilic–Superoleophilic Strategy

As mentioned earlier, superhydrophilic–submerged superoleophobic surfaces are essentially superhydrophilic–superoleophilic surfaces, i.e., amphiphilic surfaces. The expansion of the application of amphiphilic membranes led to the development of a strategy in which the filtration term is actively selected by pre-wetting the membrane surface.

For instance, Xiong et al. [48] and Tao et al. [49] prepared amphiphilic membranes that are oleophobic in water and hydrophobic in oil. Oil–water separation can be achieved by pre-wetting the membrane with either the oil or aqueous phase. However, the amphiphilic strategy faces the common issue that the separation capacity decreases when the wetting solution dries.

### 3.4. Responsiveness

The wettability of responsive separation membrane surfaces can be altered under various external stimuli, such as pH, temperature, electric potential, light, and gas. This enables the conversion between “hydrophobic” and “oleophobic” states [50,51]. However, from the perspective of membrane surface wettability during the separation process, there is no fundamental difference between the separation principles and strategies of “water-blocking” and “oil-blocking” and those of superhydrophobic–superoleophilic and superhydrophilic–superoleophobic membranes.

The aforementioned strategies are the most widely studied in oil–water membrane separation, and similar classifications are used in most review studies [36,44,45,52,53]. However, the separation strategies can be further expanded by comprehensively analyzing the separation processes of liquid-phase adsorption, passage through the pores, and detachment from the pores. In summary, the advantages and disadvantages of different separation membranes are summarised in Table 2.

### 3.5. Janus Membrane

Janus membranes, characterized by their immersion asymmetry, have attracted significant attention due to their ability to spontaneously and directionally transport liquids [16,20,54,55]. Initially considered unsuitable for oil/water separation in principle [56,57], their unique properties—different from those of conventional membranes—have led to a gradual increase in research on oil–water emulsion separation in recent years.

**Figure 11:**
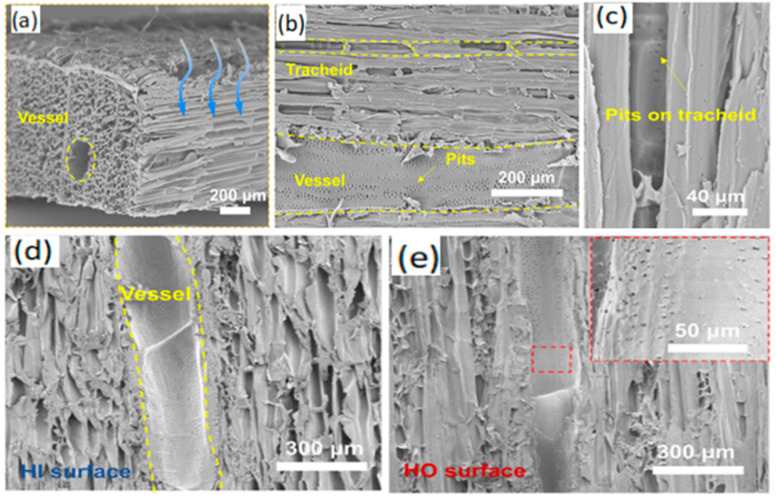
SEM image of Janus membrane structure [64]. (**a**,**b**) SEM image of the JW membrane with 3D interconnected porous structure and oriented vessel and tracheid channels. (**c**) Magnified SEM image of the microchannels showing pits on the tracheid. (**d**,**e**) SEM images of the HI and HO surface of the JW membrane.

For example, Song [58] utilized a non-solvent induced phase separation (NIPS) process to prepare a PVDF/DA/PEI mixture from a polyester nonwoven fabric. This process allowed PDA/PEI to migrate to the membrane surface, forming an asymmetric Janus membrane capable of effective oil–water separation. Yang [59] used dopamine (DA)-modified polyethylene terephthalate (PET)/PTFE composite microfiltration membranes and obtained Janus membranes by peeling off the top dense skin layer with transparent tape. This enabled the collection of oil from oil spills and oil-in-water emulsions. Zhang [60] prepared polyaniline-silica nanoparticle (PANI-SiNP)-modified Janus membranes through a dip-coating-spraying method, which could be used for the separation of oil-in-water and water-in-oil emulsions. Ding [61] modified hydrophilic ceramic membranes with cetyltrimethoxysilane (HDTMS) and subjected them to O_2_/N_2_ plasma etching to obtain Janus membranes, enabling the separation of W/O and O/W emulsions. An [62] deposited hydrophilic and positively/negatively charged carbon nanotubes (CNTs) onto hydrophobic microfiltration membranes via vacuum filtration, controlling the thickness of the hydrophilic and hydrophobic layers to regulate membrane permeability and improve oil–water emulsion separation efficiency. Wu et al. [63] achieved Janus membranes by infiltrating and removing paraffin, allowing polydopamine (PDA) to deposit only on one side of the PVDF membrane, exhibiting high separation efficiency for both oil-in-water and water-in-oil emulsions. Che et al. [64] obtained Janus membranes by spraying fluorine-containing particles on the surface of a wood membrane (Figure 11). The separation efficiency of the Janus membrane was higher than 99% for both oil-in-water and water-in-oil emulsions. Cheng et al. [65] prepared Janus membranes by growing superhydrophilic nanocoatings in situ on PVDF membranes, with separation efficiencies for oil-in-water emulsions exceeding 99.5%. Yan et al. [66] fabricated Janus polyacrylonitrile/carbon nanotube nanofiber membranes via sequential electrostatic spinning, achieving an oil-in-water emulsion separation efficiency greater than 99.8%. Huang [67] decorated CNTs onto one side of a polydopamine (PDA)/acidified carbon nanofiber (ACNTs)/polyurethane (TPU) nanofiber composite membrane, forming a PDA/ACNTs@TPU-CNTs@TPU composite membrane that enabled high-efficiency and high-throughput separation of oil-in-water and water-in-oil emulsions with excellent recyclability.

Yang [68] coated TiO_2_@PPS membranes with hydrophobic PFDS and prepared Janus membranes with switchable oil/water emulsion separation performance, achieving separation efficiencies higher than 98% for both surfaces. Wang et al. modified one side of the fabric with polydimethylsiloxane (PDMS) as the hydrophobic side and poly (N,N-Dimethylaminoethyl methacrylate) [69,70] or nonionized polyester containing oligo(ethylene glycol) monolaurate (EL) chains [71] as the hydrophilic side on the other side. These Janus membranes were prepared for separating oil–water emulsions with active agents. Zhang et al. [72] anchored hydrophilic β-FeOOH on polyvinylidene fluoride-hexafluoropropylene copolymer (PVDF-HEP) fibers using an in situ mineralization technique, obtaining Janus membranes that separated water-in-oil emulsions containing surfactants with an oil-in-oil emulsion separation efficiency greater than 99.8%. A comparison of the different Janus membranes is shown in Table 3.

Additionally, membranes designed to mimic the microstructure of the back of the desert beetle, with superhydrophobic-superoleophilic bumps on a submerged superoleophobic membrane [73,74,75], or a membranes with a structure similar to that of the desert beetle but with opposite wettability [76], can efficiently separate oil–water emulsions. The separation principles and strategies of these membranes are the same as those of Janus membranes.

### 3.6. Oil–Water Super-Double Evacuation

The superhydrophobic–superoleophilic strategy is susceptible to oil contamination, while the superhydrophilic–underwater superoleophobic strategy is essentially amphiphilic and similarly prone to contamination. The superhydrophilic–superoleophobic strategy, on the other hand, requires a stimulus response and has a long response time. All of these strategies primarily focus on the membrane surface or the inner surface of the pores. However, if the focus is shifted to the separation process from the detached pores, oil–water separation can be achieved through the oil–water super-double hydrophobicity strategy.

Zhang [28] sprayed grafted fluoride solid particles onto the surface of a stainless steel mesh membrane to obtain an oil–water double hydrophobic membrane. By applying pressure to the oil–water mixture interface, the mixture comes into contact with the auxiliary screening liquid on the lower surface. The same type of liquid as the auxiliary screening liquid can break through the mesh holes and flow freely, while different types of liquids are restricted to the top of the mesh membrane, effectively achieving the separation of oil and water. Pan et al. [77] modified the surface with fluoride to grow an ultra-double hydrophobic membrane, obtaining a fluoride-modified surface-grown copper nanopin mesh. This setup can remove both water and oil with a separation efficiency greater than 99.8% by controlling the auxiliary screening liquid.

The oil–water super-double hydrophobicity strategy addresses the disadvantage of membrane susceptibility to contamination but requires the application of a certain pressure, which limits its research scope. Nonetheless, it provides valuable insight into the influence of membrane wettability at different positions on the selectivity of oil and water during the separation process.

By expanding on the above principles and separation strategies, it is evident that the removal of oil or water does not solely correspond to the wettability of a specific area on the membrane. Rather, it is determined by the combined effects of adsorption on the membrane surface, the passage of liquid through the pore space, and the detachment process of the liquid from the membrane, each independently influenced by the wettability of different locations.

### 3.7. Multifunctional Layered Structures and Hybrid Membranes

The design and preparation of multifunctional layered structures and hybrid membranes provide innovative ideas for solving the problem of efficient separation of stable emulsions and mitigating membrane contamination. Scaffaro et al. [78] fabricated a biomimetic “branch–leaf” structure, in which graphene oxide (GO) is used as a hydrophilic ‘leaf’ and PCL fibers as a hydrophobic “branch” to realize the directional guidance of the oil–water separation path (oil adsorption on PCL, water diffusion on GO). This multifunctional hierarchical membrane (PCLGO), which also has good hydrophilic/lipophilic properties, is able to rapidly process oil-in-water emulsions (99.8% efficiency). Due to its high specific surface area, it can provide abundant adsorption sites to enhance the capture and agglomeration efficiency of emulsion droplets, maintain >99% separation efficiency after 20 cycles, be structurally stable and not easily clogged, and can effectively reduce the cleaning frequency of the membrane.

Wang et al. [79] fabricated 3D hybrid graphene macrostructures (3D GBMs). Through the three-dimensional porous network structure, the continuous flow filtration mode was realized to avoid the clogging problem of traditional membranes and enhance the emulsion treatment capacity. However, it still has problems of high production cost and environmental pollution. In summary, the comparison of hybrid and multifunctional technologies is shown in Table 4.

## 4. Conclusions

This paper analyzes the independent influence of wettability of oil-water separation membrane in the processes of surface adsorption, liquid passing through the pores and detaching from the pores; modularizes the separation process; clarifies the comprehensive mechanism of the separation effect of the wettability of membrane at different positions; summarizes the separation principle and design strategy of a special wettability oil–water separation membrane; provides theoretical guidance for the directional design of membrane materials; and helps to solve the problems of membrane contamination, pore clogging, and insufficient mass-transfer efficiency.

Future research should focus on the following directions: first, the promotion of the integration of multifunctional layered structures, through the design of biomimetic layered or hybrid membrane structures (such as the “branch–leaf” structure) and the use of different layers of selective adsorption and directional transport functions, to improve the separation efficiency of stable emulsions and alleviate membrane contamination; and second, the use of advanced manufacturing technologies, such as wet electrospinning technology, to improve the separation efficiency of oil–water membrane separation and reduce membrane contamination. The second will involve the adoption of advanced manufacturing technology, such as wet electrospinning technology, to precisely regulate the microstructure and pore distribution of membranes and realize the large-scale preparation of high-performance membranes, taking into account the structural stability and separation performance. Finally, the third research direction will involve developing an adsorption–filtration coupling strategy which combines high-efficiency adsorption sites with selective filtration pore space, and through the synergistic effect of adsorption of emulsion droplets and then high-efficiency filtration and separation, this will be able to break through the limitations of the traditional single mode of separation and improve the separation efficiency and anti-pollution ability of complex oil–water systems. These directions will provide key support for the industrialized application of special infiltration oil–water membrane separation, and promote these strategies to play a greater role in the actual treatment of oily wastewater.

## Figures and Tables

**Table 1:** Inadequateness of these reviews.

References	Inadequateness
Jiang et al. [2,3,4]	Absence of dynamic wetting theory Limitations of the Wenzel/Cassie model
Tian et al. [5]	Lack of harmonized anti-wear test standards
	Lack of data support for long-term durability
Milionis et al. [6]	Limited adsorption capacity for high viscosity oils Poorly environmentally friendly
Zhang et al. [7] Liang et al. [8,9,10,11,12]	Unsummarized separation strategy for oil–water membrane separation

**Table 2:** Comparison of different membrane studies.

Separation Strategy	Reference	Contact Angle	Advantage	Disadvantage	Typical Substrate Material
Superhydrophobic–Superoleophilic	Feng [1]; Rasouli [36]	WCA > 150° (e.g., 156.2° ± 2.8°), OCA ≈ 0°	Initially, it was mainly used for light oil separation (density < water)	Easily clogged by oil contamination Requires additional energy consumption High maintenance costs	Metal mesh, ceramic, fabric
Superhydrophilic– Superoleophobic	[26,46,47]	Superoleophobic under water: WCA < 5°, OCA under water ≥ 150°; superoleophobic in air: WCA ≈ 0°, OCA ≥ 150°	Underwater oleophobic; anti-pollution; easy to clean Superoleophobic in the air Can block oil and permeability	Underwater superoleophobicity: amphiphilic surface (oil penetration when water-free) Slow response to infiltrative transitions	Metal mesh, polymer film, electrospun film
Superhydrophilic–underwater superoleophobic	Zarghami [43]	WCA < 5°, underwater OCA ≥ 150°	It is highly resistant to pollution and can be recycled	Failure in a waterless environment; can allow oil droplets to penetrate pores	Same as superhydrophilic–superolepophobic
Superhydrophilic–superoleophilic	Xiong [48] Tao [49]	Switchable wettability: hydrophobic (oil-pre-soaked)/oleophobic (water-pre-soaked).	High environmental adaptability Active filter phase selection	Dried state: reduced separation capacity (prewetted solution)	Metal mesh, composite film
Responsive	[36,44,45,50,51,52,53]	Switchable to “Water Block” or “Oil Block”	Flexibly adapt to different separation scenarios	Response speed, cycle stability and cost issues	Smart materials (e.g., temperature-sensitive/photopolymer-sensitive)

**Table 3:** Comparison of Janus membrane oil–water separation efficiency.

Researcher	Separation Efficiency
Zhang [60]	>99.8%
Cheng [65]	>99.5%
Yan [66]	>99.8%
Che [64]	>99%

**Table 4:** Hybrid and multifunctional techniques: comparative table.

	3D GBMs	PCLGO
Flux	High	Extremely high
Rejection rate	>99% (adsorption-based)	99.8% (emulsion separation)
Fouling resistance	Electrochemical regeneration (MB degradation)	Petroleum ether cleaning and regeneration
Suitability for stable emulsions	Requires assisted emulsion breaking (e.g., electrochemical or photocatalytic)	Designed for emulsions
